# Talquetamab-associated hypokalemia

**DOI:** 10.1093/ckj/sfag368

**Published:** 2026-10-21

**Authors:** Krishna Mohita Kuruvada, Hassane Izzedine, Rimda Wanchoo, Kenar D Jhaveri

**Affiliations:** Division of Kidney Diseases and Hypertension, Northwell Health and Donald and Barbara Zucker School of Medicine at Hofstra/Northwell, Great Neck, NY, USA; Department of Nephrology, Peupliers Private Hospital, Paris, France; Division of Kidney Diseases and Hypertension, Northwell Health and Donald and Barbara Zucker School of Medicine at Hofstra/Northwell, Great Neck, NY, USA; Division of Kidney Diseases and Hypertension, Northwell Health and Donald and Barbara Zucker School of Medicine at Hofstra/Northwell, Great Neck, NY, USA

To the Editor,

T-cell–redirecting therapies have transformed the treatment of relapsed or refractory multiple myeloma. The MonumenTAL-3 trial by Mina *et al*. [1] demonstrates the efficacy of talquetamab-based combinations while highlighting treatment-related electrolyte abnormalities, including hypokalemia. Hypokalemia occurred in 20%−25% of patients in the talquetamab-containing arms versus 16% in the daratumumab–pomalidomide–dexamethasone control arm, suggesting a direct or indirect contribution from talquetamab to potassium loss.

This observation is consistent with previous talquetamab trials, including those enrolling patients with extramedullary disease (Table 1) [2, 3]. Although often dismissed as minor, hypokalemia is far from benign; severe or sustained depletion predisposes patients to muscle weakness, fatigue, and potentially life-threatening arrhythmias. Because many myeloma patients have cardiovascular comorbidities or receive other cardiotoxic therapies, maintaining electrolyte balance is particularly important.

**Table 1:** Percentage of hypokalemia or decreased potassium associated with talquetamab therapy.

Study or trial	Treatment regimen	Hypokalemia or decreased potassium (%)		Ref.
		Any grade	Grade ≥3	
MonumenTAL-1	Talquetamab monotherapy	31.0	4.4	[2]
MonumenTAL-3	Talquetamab–daratumumab–pomalidomide	24.6	6.2	[1]
MonumenTAL-3	Talquetamab–daratumumab	20.1	3.6	[1]
RedirecTT-1	Talquetamab plus teclistamab	30.0	8.0	[3]

The mechanism of talquetamab-associated hypokalemia remains unclear. Talquetamab targets G-protein coupled receptor class C group 5 member D (GPRC5D) on plasma cells and CD3 on T cells, but no direct renal tubular or gastrointestinal on-target effect of GPRC5D has been established [4], making off-tumor toxicity an unlikely sole explanation. The prescribing information reports decreased sodium and phosphate in ∼31% and 44% of patients, respectively, suggesting a generalized disturbance in electrolyte homeostasis rather than an isolated defect in potassium handling [5].

Several overlapping mechanisms may contribute. Cytokine release syndrome (CRS) can alter renal hemodynamics and vascular permeability, and cytokine-mediated tubular dysfunction may transiently impair electrolyte reabsorption. Inflammatory stress also drives catecholamine release, promoting intracellular potassium shifts independent of true losses. Gastrointestinal toxicities, including subclinical diarrhea and reduced oral intake from dysgeusia, may compound deficits. Concomitant diuretics, corticosteroids, or laxatives may further contribute.

As talquetamab becomes integrated into standard practice, proactive electrolyte monitoring is vital. Baseline and serial monitoring of potassium, sodium, and phosphate should be mandated during initial cycles, during the highest-risk window for CRS. Translational studies are needed to determine whether wasting reflects intracellular shifts, transient tubular injury, or gastrointestinal loss—mechanisms that are not mutually exclusive and may vary with disease burden, CRS severity, nutritional status, renal function, and concomitant therapy. Clarifying these pathways will enable targeted mitigation, ensuring that talquetamab’s efficacy is not compromised by preventable metabolic complications.

## References

[1] Mina R, Beksac M, Rodríguez-Otero P et al. Talquetamab–daratumumab in relapsed or refractory myeloma. N Engl J Med;2026;395:671–83. 10.1056/NEJMoa2604657 42294841

[2] Chari A, Minnema MC, Berdeja JG et al. Talquetamab, a T-cell–redirecting GPRC5D bispecific antibody for multiple myeloma. N Engl J Med. 2022;387:2232–44. 10.1056/NEJMoa2204591 36507686

[3] Kumar S, Mateos M-V, Ye JC et al. Dual targeting of extramedullary myeloma with talquetamab and teclistamab. N Engl J Med. 2026;394:51–61. 10.1056/NEJMoa2514752 41358582

[4] Jiang X, Xu X, Wu M, Teng L et al. GPRC5A: an emerging biomarker in human cancer. Biomed Res Int. 2018;2018:1. 10.1155/2018/1823726 PMC6207857 30417009

[5] U.S. Food and Drug Administration . Talquetamab prescribing information. DailyMed, (Date accessed 15 September 2026).

